# PET Tau Imaging and Motor Impairments Differ Between Corticobasal Syndrome and Progressive Supranuclear Palsy With and Without Alzheimer's Disease Biomarkers

**DOI:** 10.3389/fneur.2020.00574

**Published:** 2020-07-10

**Authors:** Anna Vasilevskaya, Foad Taghdiri, Namita Multani, Cassandra Anor, Karen Misquitta, Sylvain Houle, Charles Burke, David Tang-Wai, Anthony E. Lang, Susan Fox, Elizabeth Slow, Pablo Rusjan, Maria C. Tartaglia

**Affiliations:** ^1^Institute of Medical Science, University of Toronto, Toronto, ON, Canada; ^2^Tanz Centre for Research in Neurodegenerative Diseases, University of Toronto, Toronto, ON, Canada; ^3^Division of Neurology, Toronto Western Hospital, University Health Network, Toronto, ON, Canada; ^4^PET Centre, Centre for Addiction and Mental Health, Toronto, ON, Canada; ^5^School of Medicine and Dentistry, Western University, Windsor, ON, Canada; ^6^Edmond J. Safra Program for Parkinson Disease and the Morton and Gloria Shulman Movement Disorders Clinic, Toronto Western Hospital, University Health Network, Toronto, ON, Canada

**Keywords:** corticobasal syndrome, progressive supranuclear palsy, Alzheimer's disease, positron emission tomography, motor symptoms, PSPRS, UPDRS

## Abstract

**Introduction:** Frontotemporal lobar degeneration (FTLD)-related syndrome includes progressive supranuclear palsy (PSP) and corticobasal syndrome (CBS). PSP is usually caused by a tauopathy but can have associated Alzheimer's disease (AD) while CBS can be caused by tauopathy, transactive response DNA binding protein 43 kDa, or AD pathology. Our aim was to compare the parkinsonian syndromes presenting without AD biomarkers (CBS/PSP-non-AD) to parkinsonian syndromes with AD biomarkers (CBS/PSP-AD).

**Materials and Methods:** Twenty-four patients [11 males, 13 females; age (68.46 ± 7.23)] were recruited for this study. The whole cohort was divided into parkinsonian syndromes without AD biomarkers [*N* = 17; diagnoses (6 CBS, 11 PSP)] and parkinsonian syndromes with AD biomarkers [*N* = 7; diagnoses (6 CBS-AD, 1 PSP-AD)]. Anatomical MRI and PET imaging with tau ligand [18F]-AV1451 tracer was completed. Cerebrospinal fluid analysis or [18F]-AV1451 PET imaging was used to assess for the presence of AD biomarkers. Progressive supranuclear palsy rating scale (PSPRS) and unified Parkinson's disease rating scale (UPDRS) motor exam were implemented to assess for motor disturbances. Language and cognitive testing were completed.

**Results:** The CBS/PSP-non-AD group [age (70.18 ± 6.65)] was significantly older (*p* = 0.028) than the CBS/PSP-AD group [age (64.29 ± 7.32)]. There were no differences between the groups in terms of gender, education, years of disease duration, and disease severity as measured with the Clinical Dementia Rating scale. The CBS/PSP-non-AD group had significantly lower PET Tau Standard Volume Uptake Ratio (SUVR) values compared to the CBS/PSP-AD group in multiple frontal and temporal areas, and inferior parietal (all *p* < 0.03). The CBS/PSP-non-AD group had significantly higher scores compared to the CBS/PSP-AD group on PSPRS (*p* = 0.004) and UPDRS motor exam (*p* = 0.045). The CBS/PSP-non-AD group had higher volumes of inferior parietal, precuneus, and hippocampus (all *p* < 0.02), but lower volume of midbrain (*p* = 0.02), compared to the CBS/PSP-AD group.

**Discussion:** The CBS/PSP-non-AD group had higher motor disturbances compared to the CBS/PSP-AD group; however, both groups performed similarly on neuropsychological measures. The AD biomarker group had increased global uptake of PET Tau SUVR and lower volumes in AD-specific areas. These results show that the presenting phenotype of CBS and PSP syndromes and the distribution of injury are strongly affected by the presence of AD biomarkers.

## Introduction

Frontotemporal lobar degeneration (FTLD) is an umbrella term used to describe neurodegenerative diseases with an underlying pathology preferentially involving frontal and temporal lobes (1). Two syndromes that fall under the FTLD umbrella are corticobasal syndrome (CBS) and progressive supranuclear palsy (PSP). CBS and PSP are syndromes with motor and cognitive deficits with high overlap in clinical presentation and underlying pathology, but with their own distinct features (2, 3). CBS presents with subcortical motor features of akinetic rigidity, dystonia, and progressive asymmetric bradykinesia, as well as cortical deficits of ideomotor limb apraxia, myoclonus, and alien limb phenomenon (4–6). Cognitive features that are prominent in CBS include language disfunction (i.e., non-fluency, word finding difficulty, and sentence repetition problems), visuospatial, and social cognition abnormalities. Executive function and memory impairments may present in some CBS cases, but are non-specific and cannot distinguish CBS from other neurodegenerative diseases like PSP or Alzheimer's disease (AD) (3). Initially, the pathology underlying CBS was thought to be exclusively corticobasal degeneration (CBD)—a 4-repeat tauopathy. With the emergence of multiple post-mortem case studies, CBS is now known to have heterogeneous neuropathological underpinnings including AD, Pick's disease, FTLD with transactivation response DNA binding protein 43 kDa (TDP-43) inclusions, and FTLD with ubiquitin-immunoreactive inclusions negative for TDP-43 (3, 7–9) as well as cerebrovascular pathology (3, 9, 10). The most common neuropathological cause of CBS is due to CBD; however, it accounts for <50% of all CBS cases. The second and third most common causes of CBS are PSP and AD pathology, respectively (11).

PSP is the other motor-predominant FTLD syndrome and is associated with early postural instability, falls, and abnormal eye movements (12, 13). This describes the classic Richardson's syndrome; it is now known that PSP can present with language, cognitive, or behavioral deficits (14). The underlying pathology of PSP (a 4-repeat tauopathy, like CBD) is confirmed in ~90% of all cases of the Richardson syndrome and consists of 4-repeat tau immunoreactive inclusions in brainstem and basal ganglia (12).

AD is a neurodegenerative disease associated with abnormal deposits of amyloid β-peptide (Aβ) plaques and neurofibrillary tangles of hyperphosphorylated tau protein and is a mixed 3-repeat and 4-repeat tauopathy with tau in the form of paired helical filaments. It is now known that AD can present with a variety of phenotypes so that aside from the classic amnestic variant, there can be presentations with language, visual processing, executive, and behavioral symptoms (15). There is also the increased recognition that co-pathology exists and is a common feature of neurogenerative diseases (9, 16, 17). It is unclear how the presence of AD biomarkers as a surrogate of AD pathology affects the clinical presentation of FTLD-motor syndromes. The aim of the current study is to compare the parkinsonian syndromes presenting without AD biomarkers to parkinsonian syndromes with AD biomarkers on measures of motor scales, cognition, and neuroimaging outcomes.

## Materials and Methods

### Participants

Twenty-four participants with current diagnoses of PSP or CBS were included in this study. Participants were included if they were 18–90 years old, able to read, understand, and speak English for neuropsychological testing. Participants must have had a reliable study partner who could provide independent evaluation of functioning. Exclusion criteria were history of traumatic brain injury, brain tumors, stroke, or other neurological or psychiatric disorders that could explain symptoms. The diagnosis of PSP was made based on the National Institute of Neurological Disorders and Stroke Society of Progressive Supranuclear Palsy (NINDS-SPSP), and further refined in the multicenter on Neuroprotection and Natural History in Parkinson Plus Syndromes (NNIPPS) study (12, 18, 19). The diagnosis of CBS was made based on the current criteria for CBS (20). Nineteen of twenty-four participants underwent a lumbar puncture procedure to collect and analyze cerebrospinal fluid (CSF) for the presence of AD pathology. For the five participants who refused lumbar punctures, PET tau imaging was examined by a cognitive neurologist (MCT) for evidence of a pattern typical of AD. Participants were divided into two groups: (1) participants with diagnoses of PSP and CBS negative for AD biomarkers (FTLD-non-AD) and (2) participants with diagnoses of PSP and CBS positive for AD biomarkers (FTLD-AD). Written consent was obtained from all study participants and caregivers. The study was approved by the University Health Network and Center for Addiction and Mental Health Research Ethics Boards.

### Motor and Neuropsychological Testing

All participants were assessed by a cognitive neurologist (MCT), and Progressive Supranuclear Palsy Rating Scale (PSPRS) (21) and Unified Parkinson's Disease Rating Scale (UPDRS) (22) motor part were completed. The global level of functioning was measured using clinical dementia rating scale (CDR) (23) through interviews of caregivers by trained research assistants. CDR global score and sum of boxes were calculated. Participants underwent comprehensive neuropsychological testing across the following domains: language, executive function, memory, and visuospatial function. Language assessments included Multilingual Naming Test (MiNT) (24), Pyramids and Palm Trees (25), sentence repetition, and semantic and lexical fluency (26). Executive function assessments included trail making test part B (TMT B) (27, 28) and Digit Symbol coding test (29). Memory assessments included California Verbal Learning Test learning score, delayed 10-min recall scores, and Benson figure recall score. Attention and working memory are assessed using digit span forward and digit span backward, respectively (26, 29). Assessment of visuospatial function was completed using the Visual Object and Space Perception battery (VOSP) (30). Higher score for TMT part B signified worse function, while for the rest of the assessments, the higher score signified better cognitive functioning.

### Cerebrospinal Fluid

Lumbar puncture procedure to collect CSF was performed following the AD Neuroimaging Initiative (ADNI) protocol (31). CSF was collected into polypropylene tubes, and a sandwich ELISA technique was implemented to measure levels of Aβ42, phosphorylated tau (p-tau), and total tau (t-tau) (32, 33). AD pathology was deemed present if p-tau > 68 pg/ml and Aβ42 to t-tau index <0.8 (34, 35).

### MRI Acquisition

All structural and DTI scans were obtained using a 3-T MRI Scanner (GE Signa HDx, Milwaukee, WI, USA) with 8-channel head coil. T1-weighted structural MRI scans were acquired using inversion recovery spoiled gradient echo (IR-SPGR) in the sagittal plane using the following scan parameters: TE = 2.8 ms, TR = 7 ms, flip angle = 11°; 176 slices, slice thickness = 1.2 mm, 256 × 256 matrix, FOV = 26 cm. One DWI scan was obtained with the diffusion gradient applied across 60 spatial directions (*b* = 1,000 s/mm^2^) and 10 non-diffusion-weighted Bo scans. The DWI was acquired with the following parameters: 2.4-mm-thick axial slices, TR = 17,000 ms, FOV = 23 cm, 2.4 × 2.4 mm in-plane resolution.

### Structural MRI Analysis

T1-weighted structural 3D MRI images analysis was performed with the FreeSurfer v.6 image analysis suite, which is freely available online with documentations (http://surfer.nmr.mgh.harvard.edu/). Structural MRI was preprocessed using the standardized “recon-all” FreeSurfer pipeline, which is described elsewhere (36). Volumes of interest for the following brain regions were extracted: posterior cingulate, inferior parietal, precuneus, lateral orbitofrontal, caudal middle frontal, hippocampus, caudate, and thalamus. Brainstem subfields FreeSurfer pipeline was implemented to extract midbrain volumes (37). To account for individual differences in head size, each volume of interest was corrected for total intracranial volume (ICV) by dividing each structure's volume by ICV (volume-to-ICV ratio) (38).

### DTI Analysis

**Figure 1:**
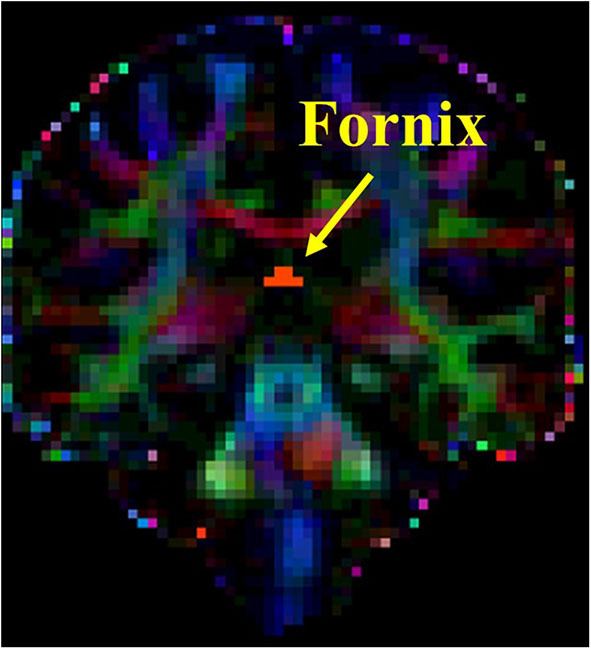
Seed placement for tractography of fornix.

The DTI analysis was conducted using the FMRIB Software Library (FSL) tools (http://www.fmrib.ox.ac.uk/fsl/fdt/index.html). The region of interest (ROI) analysis was performed for the following tracks: right and left superior longitudinal fasciculus (SLF) and fornix. Multiple DTI metrics representing different aspects of white matter integrity were extracted including the following: (a) fractional anisotropy (FA), (b) medial diffusivity (MD), (c) axial diffusivity (AxD), and (d) radial diffusivity (RD). The processing steps of the DTI data, ROI definition, and fiber tracking steps for SLF were completed as previously described (39, 40). The ROI for the fornix was placed on the coronal slice at the point where the posterior pillars of the fornix join together to form the body of fornix (see Figure 1). Prior to conducting DTI analysis, subjects' FLAIR images were reviewed by a neurologist (MCT) for presence of no or minimal amount of white matter hyperintensity to ensure accurate tractography results.

### PET Acquisition and Analysis

All participants underwent PET imaging with 5 mCi of [F-18]AV1451 ([F-18]T807; Flortaucipir, AVID Radiopharmaceuticals) tau-specific tracer. Twenty-one participants were scanned using a Biograph HiRez XVI PET/CT scanner (Siemens Molecular Imaging, Knoxville, TN, USA), while three participants were scanned using High-Resolution Research Tomograph (HRRT) (CPS/Siemens, Knoxville, TN, USA) PET scanner.

Emission PET data were acquired in list mode for 75 min starting 45 min after a bolus injection of [F-18]AV1451. The emission list mode data were re-binned into eight 3D sinograms (1 × 5 min, 7 × 10 min). For the HRRT acquisitions, a transmission scan was acquired using a single photon point source, ^137^Cs (*T*_½_ = 30.2 years, *E*_γ_ = 662 keV), immediately after the emission acquisition, which was then used to correct the emission data for photon attenuation. Similarly, for the PET/CT acquisitions, a low-dose CT scan was acquired immediately prior to the emission acquisition for the attenuation correction of the emission data. The 3D sinograms were gap-filled, for the HRRT, normalized and scatter corrected, prior to Fourier rebinning of the 3D sinograms into 2D sinograms. The images were reconstructed from the 2D sinograms using a 2D filtered-back projection algorithm, with a HANN filter (HRRT) or ramp filter (PET/CT) at Nyquist cutoff frequency. During image reconstruction, the images were dead-time-corrected and decay-corrected to the start of acquisition. For the HRRT, the reconstructed image had 256 × 256 × 207 1.22-mm isotropic voxels with a reconstructed resolution of *ca*. 4.5 mm., full width at half maximum (FWHM) in-plane and axially. For the PET/CT, the image had 256 × 256 × 81 voxels each of 2 × 2 × 2.07 mm and an in-plane and axial resolution of 5 and 5.5 mm FWHM, respectively. A thermoplastic mask was made for each participant and used in conjunction with a head fixation system for the duration of both the transmission/CT acquisition and PET acquisition to constrain the subject's head movement. In-house ROMI software was implemented for ROI analysis of the PET data (41). Briefly, ROMI uses a T1-MRI of each subject and a template of ROIs based on the MNI template to individualize the ROIs to each subject's MRI. ROMI uses the segmentation from Statistical Parametric Mapping version 8 (SPM8; https://www.fil.ion.ucl.ac.uk/spm/software/spm8/) and Matlab 8.5.0 (Math Works, Natick, MA) to find the non-linear transformation and later uses the probability of each voxel to be gray matter and some morphological operations to refine the delineation. After realignment of the PET frames to correct for potential motion, a summed PET image was produced for each subject. Normalized mutual information implemented in Statistical Parametric Mapping version 2 (SPM2; https://www.fil.ion.ucl.ac.uk/spm/software/spm2/) was used to co-register the PET and MRI images. The rigid-body transformation was applied to the individualized ROIs to map them into the PET image. The PET images were corrected for partial volume effect (42). ROIs in the PET space were used to mask the PET images and extract the time activity curves for each ROI. For each ROI (i.e., dorsal caudate, insula, thalamus, midbrain, lateral temporal, prefrontal cortex, dorsolateral prefrontal cortex, ventrolateral prefrontal cortex, orbitofrontal cortex, hippocampus, inferior parietal, and cortical gray matter) and time frame, standard uptake volume ratios (SUVRs) were calculated using cerebellar gray matter as the reference region. SUVR of each time frame was averaged for the frames between 50 and 80 min post-injection time.

### Voxel-Based PET Analysis

Voxel-based analysis on the PET images was completed using the Statistical Parametric Mapping version 12 (SPM12; https://www.fil.ion.ucl.ac.uk/spm/software/spm12/) and Matlab 8.5.0 (Math Works, Natick, MA). PET frames were realigned to correct for motion, and voxel intensity in each PET frame was normalized to the average signal from the inferior cerebellar gray matter, which was extracted using a native atlas from the automated in-house ROMI software (41). Parametric maps were then averaged between 50 and 80 post-injection time and spatially normalized into the Montreal Neurological Institute (MNI) template to control for variability between subjects. Images were smoothed with a FWHM 8-mm Gaussian kernel in order to increase signal-to-noise ratio. The whole-brain exploratory between-group analysis was completed using the two-sample *t*-test from SPM12 in order to compare the FTLD-non-AD and FTLD-AD groups. Both uncorrected (*p* < 0.001; extent threshold, *k* = 50) and multiple comparison corrected results are presented (FWE corrected at *p* < 0.05; extent threshold, *k* = 50).

### Statistical Analysis

Statistical analysis was completed using IBM SPSS Statistics version 24 (IBM Corp., Armonk, NY, USA). All between-group demographics, neuropsychological testing and neuroimaging comparisons were completed using Mann-Whitney *U*-test, with the type of scanner and sex comparisons completed using Fisher's exact test. Pearson partial correlations with age as a covariate were used to analyze associations between PSPRS/UPDRS motor exam scores in relation to volumes of [18-F]AV1451 PET tau areas of interest. Pearson partial correlations with age as a covariate were used to analyze the following relationships: CSF measures with cortical gray matter PET tau; right SLF white matter integrity measures with visuospatial assessments; left SLF white matter integrity measures in relation to language assessments; fornix white matter integrity measures with memory assessments; language assessments and [18-F]AV1451 PET tau SUVR in left lateral temporal and insula areas; executive function assessments and [18-F]AV1451 PET tau SUVR in the dorsolateral prefrontal cortex; learning score and delayed recall with [18-F]AV1451 PET tau SUVR in the prefrontal cortex and left hippocampus; the Benson recall and [18-F]AV1451 PET tau SUVR in the prefrontal cortex and the right hippocampus; digit forward and digit backward with [18-F]AV1451 PET tau SUVR in the prefrontal cortex and the whole hippocampus; and the visuospatial assessments with the [18-F]AV1451 PET tau SUVR in the inferior parietal area. False discovery rate (FDR) correction with Benjamini–Hochberg procedure has been applied for all multiple comparisons and both adjusted and non-adjusted *p*-values are reported with a significance level set at *p* < 0.05.

## Results

### Participant Demographics and Neuropsychological Assessments

Twenty-four participants were included in this study (age, 68.46 ± 7.23 years; disease duration, 5.63 ± 4.74 years; 11 males and 13 females). Among the cohort were 12 CBS cases with 6 CBS positive for AD biomarkers and 12 PSP cases with 1 PSP case positive for AD biomarkers. The between-group demographics on FTLD-non-AD and FTLD-AD groups are presented in Table 1. The FTLD-non-AD group was significantly older than FTLD-AD group (*p* = 0.028). There were no significant differences between groups on age of disease onset, years of education, sex, disease duration, or the PET scanner used (all *p* > 0.19). The FTLD-non-AD group had significantly higher scores on both the PSPRS (*p* = 0.004) and UPDRS motor scales (*p* = 0.045) compared to the FTLD-AD group. Finally, there was no significant difference between FTLD-non-AD and FTLD-AD groups on CDR global and CDR sum of boxes scores (all *p* > 0.3). The FTLD-non-AD and FTLD-AD groups did not differ on measures of language, executive function, memory, or visuospatial function (see Table 2). There was a trend in the FTLD-non-AD group to have higher scores in sentence repetition (unadjusted *p* = 0.031) and digit span forward (unadjusted *p* = 0.06) assessments compared to the FTLD-AD group; however, this did not survive multiple comparisons.

**Table 1:** Demographics of the FTLD-non-AD and FTLD-AD groups (mean ± standard deviation).

	**FTLD-non-AD**	**FTLD-AD**	***p*-value**
***N***	**17**	**7**	
**Demographics**			
Age (years)	70.18 ± 6.65	64.29 ± 7.32	0.028
Age of onset (years)	63.82 ± 8.10	60.43 ± 7.68	0.19
Education (years)	14.71 ± 3.41	15.57 ± 4.39	0.58
Sex	8 Males:9 Females	3 Males:4 Females	1.00
Disease duration	6.35 ± 5.23	3.86 ± 2.85	0.28
Diagnosis	11 PSP, 6 CBS	1 PSP-AD, 6 CBS-AD	N.A.
PET scanner	15 PET/CT, 2 HRRT	6 PET/CT, 1 HRRT	1.00
PSPRS	40.76 ± 18.61	18.29 ± 6.65	0.004
UPDRS motor scale	36.06 ± 21.49	15.71 ± 8.34	0.045
CDR global score	1.09 ± 0.83	0.79 ± 0.64	0.43
CDR sum of boxes	6.88 ± 5.28	4.79 ± 3.87	0.36

*Fisher's exact test and Mann–Whitney U comparison; unadjusted significance level set at p < 0.05 (two-sided)*.

**Table 2:** Neuropsychological assessments comparison of FTLD-non-AD and FTLD-AD groups (mean ± standard deviation).

	**FTLD-non-AD**	**FTLD-AD**	**Unadjusted *p***	**Adjusted *p***
***N***	**17**	**7**		
**Neuropsychological assessments**				
**Language**				
Naming	11.47 ± 3.09	8.29 ± 4.99	0.10	N.S.
Pyramids and palm trees	40.44 ± 13.79^a^	39.60 ± 22.20^b^	0.41	N.S.
Sentence repetition	4.13 ± 1.46^b^	2.43 ± 1.90	0.031	N.S.
Semantic fluency	8.06 ± 6.13	7.57 ± 4.69	0.98	N.S.
Lexical fluency	5.76 ± 4.70	7.00 ± 4.90	0.48	N.S.
**Executive function**				
TMT B total seconds	217.14 ± 100.94^c^	267.14 ± 86.93	0.15	N.S.
Total digit symbol	21.71 ± 20.00^c^	17.43 ± 17.46	0.65	N.S.
**Memory**				
Learning score	13.12 ± 6.27	8.86 ± 7.86	0.27	N.S.
Delayed recall (10 min)	3.47 ± 2.70	2.14 ± 2.27	0.26	N.S.
Benson recall	7.43 ± 5.96^c^	3.00 ± 2.24	0.10	N.S.
Digit forward	5.47 ± 2.07	3.86 ± 1.77	0.06	N.S.
Digit backward	3.53 ± 2.32	2.43 ± 1.99	0.30	N.S.
**Visuospatial function**				
VOSP position discrimination	15.94 ± 5.76	15.67 ± 8.04^a^	0.49	N.S.
VOSP number location	4.88 ± 3.59	6.00 ± 3.58^a^	0.55	N.S.

Mann–Whitney U comparisons; unadjusted significance level set at p < 0.05 (two-sided).

FDR Benjamini–Hochberg adjusted p-value; significant at p < 0.05.

N.S., not significant after multiple comparisons.

a One participant refused the assessment.

b Two participants refused the assessment.

c *Three participants did not complete the assessment because could not move their hands*.

### [18-F]AV1451 PET Tau and CSF Measures Comparison

**Figure 2:**
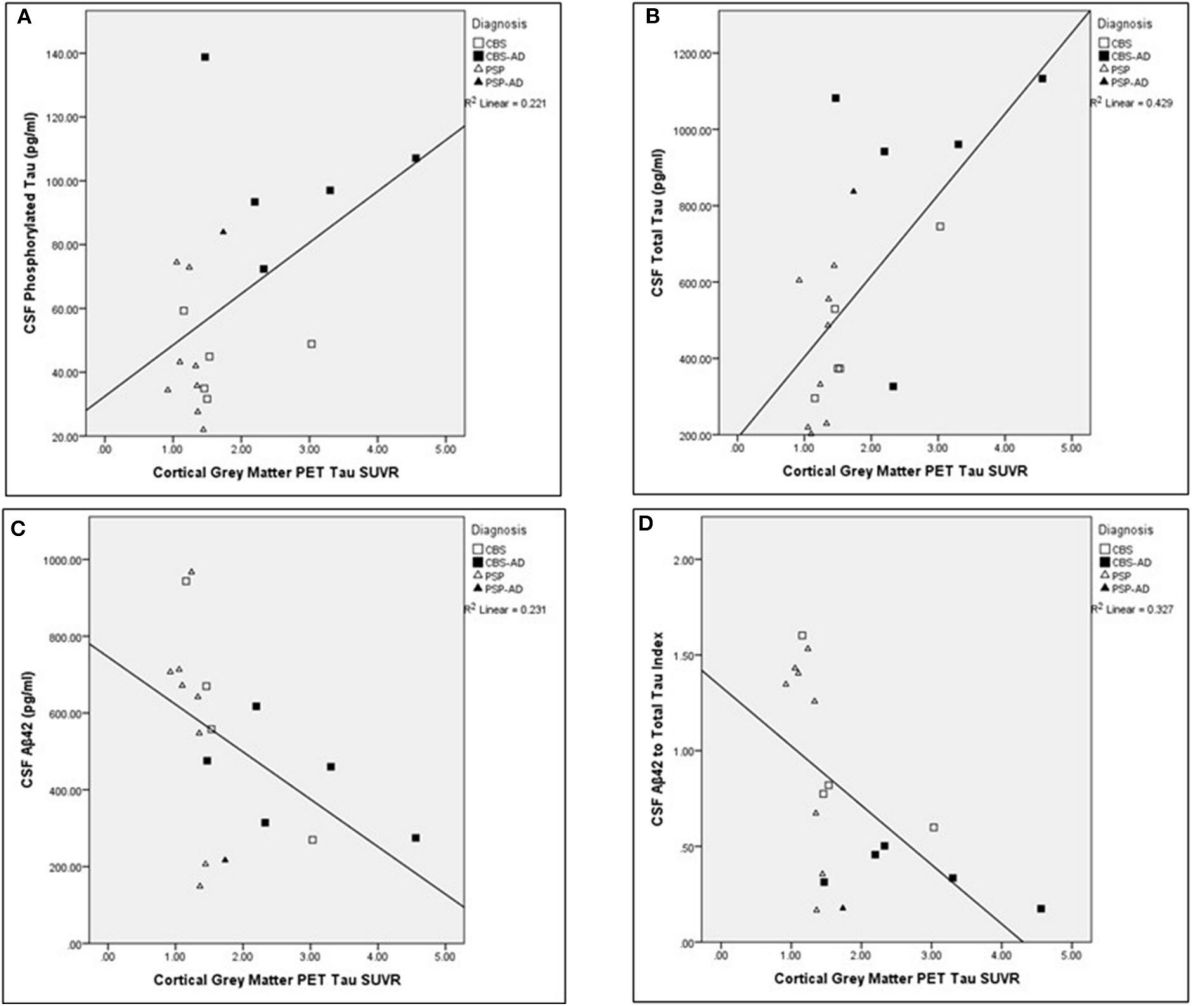
Associations between cortical gray matter PET tau SUVR values and cerebrospinal fluid analysis measures across the whole cohort. Scatter plots presenting associations between cortical gray matter PET tau SUVR and the following cerebrospinal fluid (CSF) measures: **(A)** phosphorylated tau (pg/ml; adjusted *p* > 0.1), **(B)** total tau (pg/ml; adjusted *p* < 0.05), **(C)** Aβ42 (pg/ml; adjusted *p* < 0.05), and **(D)** Aβ42 to t-tau index (adjusted *p* < 0.05). Pearson partial correlations with age as a covariate. FDR Benjamini–Hochberg adjusted *p*-values, significant at *p* < 0.05.

The associations between cortical gray matter [18-F]AV1451 PET and CSF measures were completed across the entire cohort (*N* = 19). The Aβ42 measure was not successful for one participant. Significant relationships were found between cortical PET tau SUVR values and the following CSF measures corrected for age: t-tau (pg/ml; *N* = 19; unadjusted *r* = 0.639, *p* < 0.005), Aβ42 (pg/ml; *N* = 18; unadjusted *r* = −0.557, *p* = 0.020), and ATI (*N* = 18; unadjusted *r* = −0.629, *p* < 0.01). The comparisons between cortical gray matter PET tau SUVR and t-tau, Aβ42, and ATI remained significant after controlling for multiple comparisons using FDR significant at *p* < 0.05. For visual representations of these comparisons, see Figure 2. There was no significant relationship between cortical gray matter PET tau SUVR values and p-tau (pg/ml) values (*N* = 19; unadjusted *r* = 0.388, *p* > 0.1), corrected for age.

### Neuroimaging Comparisons

Neuroimaging comparisons between FTLD-non-AD and FTLD-AD groups are summarized in Table 3. The FTLD-non-AD had significantly higher volumes compared to the FTLD-AD group in the following areas: inferior parietal (*p* = 0.018), precuneus (*p* = 0.018), and hippocampus (*p* = 0.020). The FTLD-non-AD group had significantly lower midbrain volume (*p* = 0.020), compared to the FTLD-AD group. The FTLD-non-AD and FTLD-AD groups did not significantly differ in the following volumes of interest: posterior cingulate, lateral orbitofrontal, caudal middle frontal, caudate, and thalamus. There were no significant differences between FTLD-non-AD and FTLD-AD groups in measures of white matter integrity in right and left SLF, and fornix. The FTLD-non-AD compared to the FTLD-AD group had significantly lower PET tau SUVR values in the following brain regions: lateral temporal (*p* = 0.005), dorsolateral prefrontal cortex (*p* = 0.009), ventrolateral prefrontal cortex (*p* = 0.026), orbitofrontal cortex (*p* = 0.009), hippocampus (*p* = 0.006), and inferior parietal (*p* = 0.005). There were no significant differences between FTLD-non-AD and FTLD-AD in measures of PET tau in the areas of dorsal caudate, thalamus, and midbrain.

**Table 3:** Summary of neuroimaging comparisons between FTLD-non-AD and FTLD-AD groups (mean ± standard deviation).

	**FTLD-non-AD**	**FTLD-AD**	**Unadjusted *p***	**Adjusted *p***
***N***	**17**	**7**		
**Volumetric Analysis** **(Volume-to-ICV ratio** **× ** **10**^**−3**^**)**				
Posterior cingulate	3.60 ± 0.48^a^	3.70 ± 0.60	0.42	N.S.
Inferior parietal	16.10 ± 1.80^a^	13.10 ± 1.81	0.004	***0.018***
Precuneus	12.00 ± 1.04^a^	9.70 ± 1.44	0.003	***0.018***
Lateral orbitofrontal	9.20 ± 1.46^a^	10.00 ± 0.83	0.23	N.S.
Caudal middle frontal	6.40 ± 1.47^a^	6.20 ± 0.56	0.84	N.S.
Hippocampus	5.20 ± 0.44^a^	4.60 ± 0.36	0.009	***0.020***
Caudate	4.58 ± 0.62^a^	4.45 ± 0.65	0.74	N.S.
Thalamus	7.68 ± 0.77^a^	7.81 ± 0.48	0.35	N.S.
Midbrain	3.50 ± 0.44^a^	4.00 ± 0.27	0.009	***0.020***
**DTI (mm**^**2**^**/s)**				
**Right SLF**				
FA	0.35 ± 0.03^b^	0.34 ± 0.03	0.50	N.S.
MD (× 10^−3^)	0.88 ± 0.04^b^	0.90 ± 0.05	0.46	N.S.
AxD (× 10^−3^)	1.21 ± 0.04^b^	1.23 ± 0.04	0.17	N.S.
RD (× 10^−3^)	0.72 ± 0.05^b^	0.74 ± 0.06	0.39	N.S.
**Left SLF**				
FA	0.34 ± 0.03^b^	0.34 ± 0.02	0.71	N.S.
MD (× 10^−3^)	0.89 ± 0.07^b^	0.92 ± 0.04	0.13	N.S.
AxD (× 10^−3^)	1.22 ± 0.06^b^	1.25 ± 0.05	0.12	N.S.
RD (× 10^−3^)	0.73 ± 0.08^b^	0.75 ± 0.04	0.23	N.S.
**Fornix**				
FA	0.20 ± 0.03^b^	0.20 ± 0.02	0.71	N.S.
MD (× 10^−3^)	2.16 ± 0.22^b^	2.15 ± 0.22	0.82	N.S.
AxD (× 10^−3^)	2.60 ± 0.23^b^	2.58 ± 0.26	1.00	N.S.
RD (× 10^−3^)	1.94 ± 0.22^b^	1.93 ± 0.20	0.82	N.S.
**PET (SUVR)**				
Dorsal caudate	1.52 ± 0.38	1.90 ± 0.43	0.06	N.S.
Thalamus	1.43 ± 0.24	1.64 ± 0.37	0.17	N.S.
Midbrain	1.45 ± 0.30	1.42 ± 0.26	0.87	N.S.
Lateral temporal	1.33 ± 0.43	3.12 ± 1.35	0.001	***0.005***
Dorsolateral prefrontal cortex	1.62 ± 0.57	3.22 ± 1.76	0.005	***0.009***
Ventrolateral prefrontal cortex	1.53 ± 0.46	2.77 ± 1.45	0.017	***0.026***
Orbitofrontal cortex	1.52 ± 0.39	2.53 ± 1.23	0.004	***0.009***
Hippocampus	1.20 ± 0.28	1.80 ± 0.39	0.002	***0.006***
Inferior parietal	1.49 ± 0.56	3.26 ± 1.53	0.001	***0.005***

Mann–Whitney U comparisons; unadjusted significance level set at p < 0.05 (two-sided).

FDR Benjamini–Hochberg adjusted p-values presented in bold italics; significant at p < 0.05.

N.S., not significant after multiple comparisons.

DTI, diffuse tensor imaging; SLF, superior longitudinal fasciculus; PET, positron emission tomography; SUVR, standard uptake volume ratio; FA, fractional anisotropy; MD, mean diffusivity; AxD, axial diffusivity; RD, radial diffusivity.

a Data for one participant are missing because of FreeSurfer preprocessing failure.

b *Data for three participants are missing because of movement during DTI acquisition*.

### Voxel-Based PET Analysis

**Figure 3:**
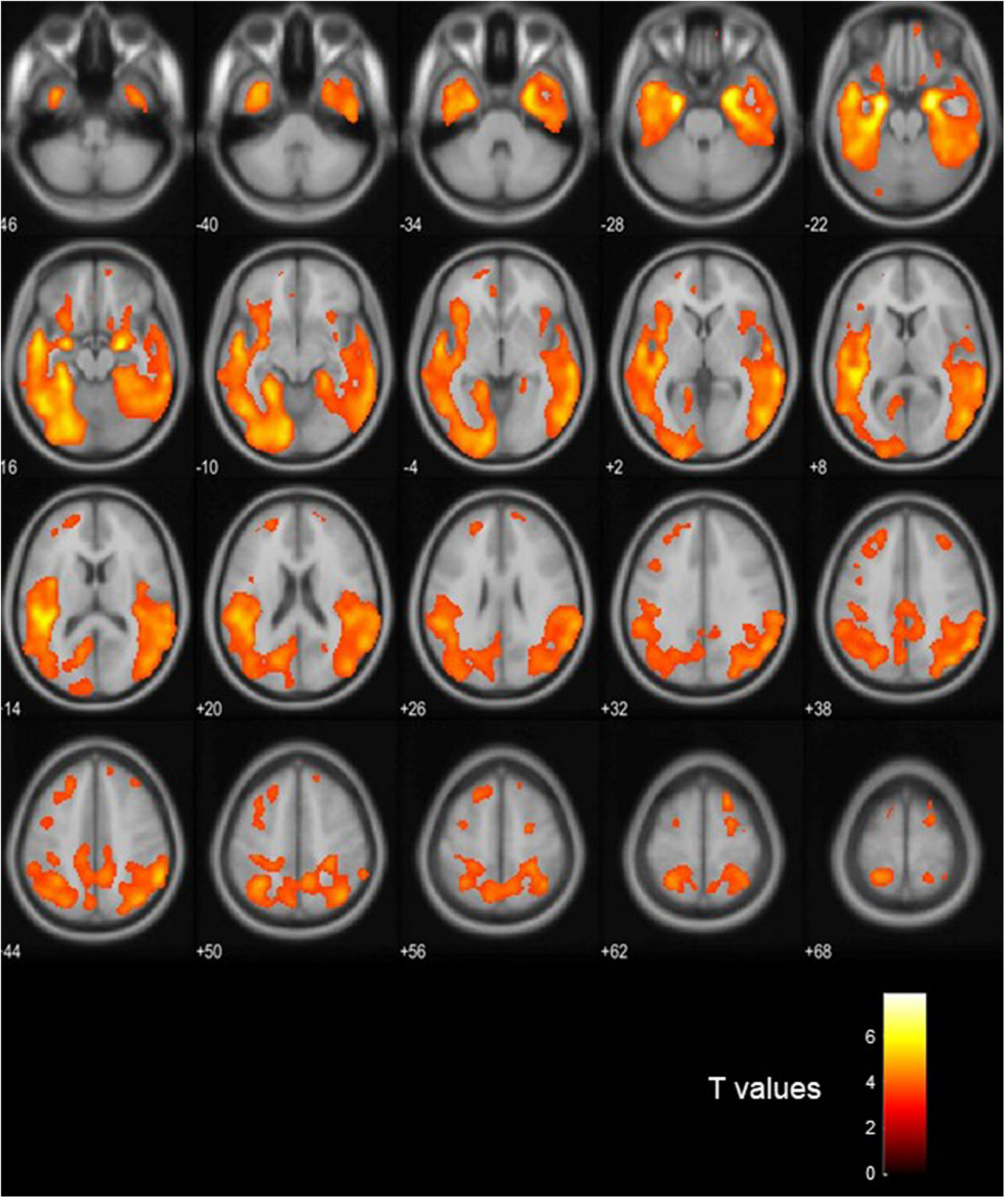
Visualization of the voxel-based [18-F]AV1451 PET tau analysis. The colors signify the regions with increased [18-F]AV1451 signal in the FTLD-AD group, compared to the FTLD-non-AD group (uncorrected *p* < 0.001; extent threshold, *k* = 50).

**Figure 4:**
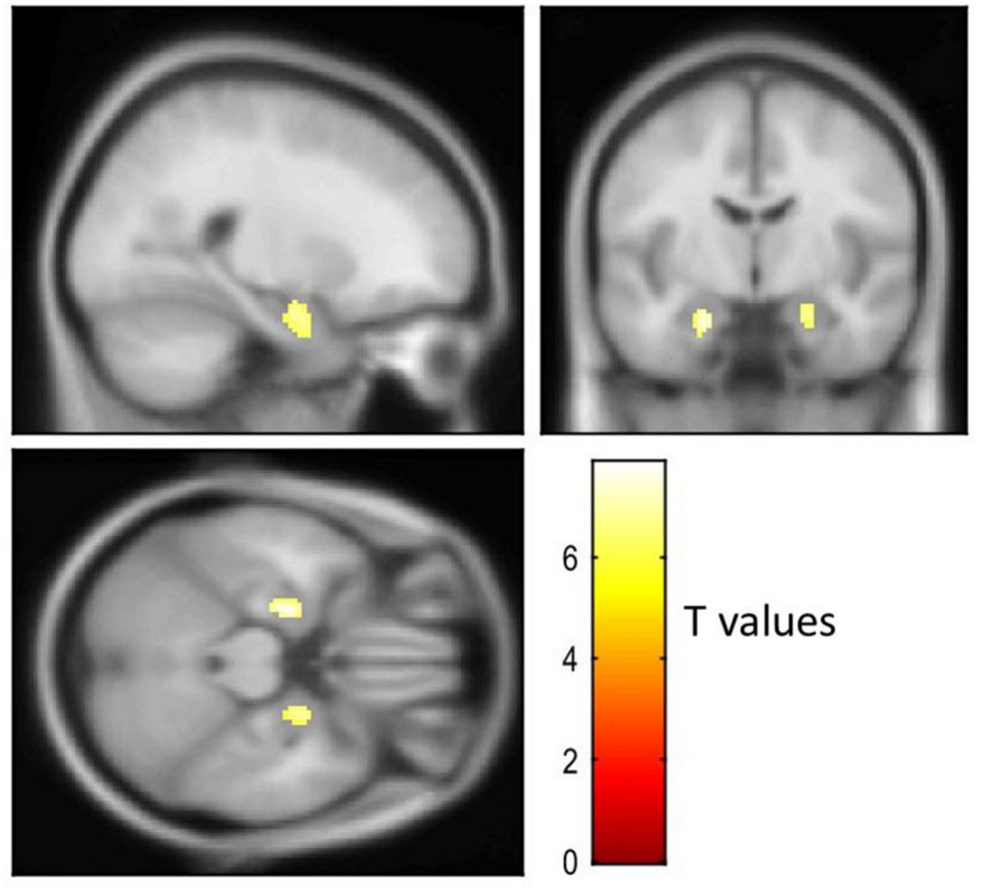
Visualization of the voxel-based [18-F]AV1451 PET tau analysis. The cluster with increased [18-F]AV1451 signal in the FTLD-AD group, compared to the FTLD-non-AD group after controlling for multiple comparisons (FEW-corrected *p* < 0.05; extent threshold, *k* = 50).

Figure 3 shows visual comparison of brain regions with higher [18-F]AV1451 PET tau signal among the FTLD-AD, compared to the FTLD-non-AD group (unadjusted *p* < 0.001; extent threshold, *k* = 50). The increased uptake in the FTLD-AD group is extensive among posterior and middle temporal regions; however, these clusters did not all survive multiple comparisons. Figure 4 shows the two clusters in the bilateral hippocampi that survived the multiple comparisons (FWE *p* < 0.05; corrected *T* = 6.02; extent threshold, *k* = 50). The bilateral hippocampi showed significantly increased uptake of [18-F]AV1451 PET tau in the FTLD-AD group in comparison to the FTLD-non-AD group.

### Relationship Between [18-F]AV1451 PET Tau and Neuropsychological Assessments

The ROI analysis exploring the associations between [18-F]AV1451 PET tau and neuropsychological assessments was completed across the entire cohort (*N* = 24). Out of the language assessments, a significant relationship was found between naming and left lateral temporal PET tau SUVR values (*N* = 24; unadjusted *r* = −0.469, *p* = 0.024), controlled for age. Three participants refused to undergo the Pyramids and Palm Trees assessment and significant relationships were found between the Pyramids and Palm Trees scores and the following areas of interest: left lateral temporal PET tau SUVR (*N* = 21; unadjusted *r* = −0.599, *p* = 0.005), controlled for age. Two participants refused the sentence repetition assessment, and there were trends between the sentence repetition scores and the following areas of interest: left lateral temporal PET tau SUVR (*N* = 22; unadjusted *r* = −0.430, *p* = 0.052), adjusted for age. The relationships between Pyramids and Palm Trees scores and left lateral temporal PET tau SUVR values remained significant after controlling for multiple comparisons using FDR significant at *p* < 0.05. There were no significant relationships between the executive function assessments and the dorsolateral prefrontal cortex PET tau SUVR, controlled for age. Out of the memory assessments, a significant relationship was found between the learning score and the left hippocampus PET tau SUVR (*N* = 24; unadjusted *r* = −0.640, *p* = 0.001), controlled for age. The delayed recall was significantly associated with the left hippocampus PET tau SUVR (*N* = 24; unadjusted *r* = −0.520, *p* = 0.011), controlled for age. Three participants could not complete the Benson figure visual recall, and there were significant relationships between the Benson recall scores and the right hippocampus PET tau SUVR (*N* = 21; unadjusted *r* = −0.630, *p* = 0.003), controlled for age. The digit forward scores had a significant association with prefrontal cortex PET tau SUVR (*N* = 24; unadjusted *r* = −0.601, *p* = 0.002), controlled for age. The digit backward scores had significant associations with the following areas of interest: prefrontal cortex PET tau SUVR (*N* = 24; unadjusted *r* = −0.608, *p* = 0.002) and hippocampus PET tau SUVR (*N* = 24; unadjusted *r* = −0.623, *p* = 0.001), controlled for age. All of the memory comparisons with PET tau SUVR remained significant after controlling for multiple comparisons using FDR significant at *p* < 0.05. Out of the visuospatial assessments, a significant relationship was found between the position discrimination and the inferior parietal PET tau SUVR (*N* = 24; unadjusted *r* = −0.569, *p* = 0.006), controlled for age. This relationship remained significant after controlling for multiple comparisons using FDR significant at *p* < 0.05. The relationships between the neuropsychological assessments and the PET tau burden could not be assessed separately in the FTLD-non-AD and FTLD-AD groups due to small sample size.

### Relationship Between [18-F]AV1451 PET Tau and PSPRS/UPDRS Scores

The ROI analysis exploring the associations between [18-F]AV1451 PET tau in relation to PSPRS and UPDRS scores was completed across the whole cohort (*N* = 24). Neither the PSPRS nor the UPDRS scores were found to be significantly associated with any of the PET tau areas of interest, controlled for age.

### Relationship Between PSPRS Scores and Volumes

For summary of associations between PSPRS scores in relation to lateral orbitofrontal, caudal middle frontal, caudate, thalamus, and midbrain volume, controlled for age, see Table 4. There were no significant relationships between PSPRS scores and volumes of lateral orbitofrontal, caudal middle frontal, caudate, thalamus, and midbrain in the FTLD-non-AD group, controlled for age. There was a trend in the FTLD-non-AD group between PSPRS scores and lateral orbitofrontal (unadjusted *r* = −0.555, *p* = 0.032), caudal middle frontal (unadjusted *r* = −0.632, *p* = 0.011), caudate (unadjusted *r* = −0.526, *p* = 0.044), and thalami (unadjusted *r* = −0.505, *p* = 0.055) volumes, adjusted for age, but the associations did not survive multiple comparisons. There were no significant relationships between PSPRS scores and lateral orbitofrontal, caudal middle frontal, caudate, thalamus, and midbrain volumes in the FTLD-AD group, controlled for age.

**Table 4:** Associations between PSPRS scores and volumes in FTLD-non-AD and FTLD-AD groups (mean ± standard deviation).

	**FTLD-non-AD**	**FTLD-AD**
***N***	**17**	**7**
**Volumetric analysis** **(volume-to-ICV ratio)**		
Lateral Orbitofrontal	*r* = −0.555, *p* = 0.032^a^ *N.S*.	*r* = 0.019, *p* = 0.98 *N.S*.
Caudal Middle Frontal	*r* = −0.632, *p* = 0.011^a^ *N.S*.	*r* = 0.829, *p* = 0.041 *N.S*.
Caudate	*r* = −0.526, *p* = 0.044^a^ *N.S*.	*r* = 0.380, *p* = 0.46 *N.S*.
Thalamus	*r* = −0.505, *p* = 0.055^a^ *N.S*.	*r* = −0.505, *p* = 0.31 *N.S*.
Midbrain	*r* = −0.322, *p* = 0.24^a^ *N.S*.	*r* = 0.175, *p* = 0.41 *N.S*.

Pearson partial correlations with age as a covariate; unadjusted significance level set at p < 0.05.

FDR Benjamini–Hochberg adjusted p-values presented in italics; significant at p < 0.05.

N.S., not significant after multiple comparisons.

a *Data for one participant are missing because of FreeSurfer preprocessing failure*.

### Relationship Between UPDRS Scores and Volumes

For summary of associations between UPDRS motor scale scores in relation to caudate, thalamus, and midbrain, controlled for age, see Table 5. There was a significant negative correlation in the FTLD-non-AD group between UPDRS motor scale scores in relation to caudate (*r* = −0.891, *p* < 0.003), controlled for age. There were no significant associations in the FTLD-non-AD group between UPDRS motor scale scores and thalami and midbrain volumes, controlled for age. There were no significant association in the FTLD-AD group between UPDRS motor scale scores and any volumes of interest (i.e., caudate, thalamus, midbrain), controlled for age.

**Table 5:** Associations between UPDRS motor scale scores and volumes in FTLD-non-AD and FTLD-AD groups (mean ± standard deviation).

	**FTLD-non-AD**	**FTLD-AD**
***N***	**17**	**7**
**Volumetric analysis** **(volume-to-ICV ratio)**		
Caudate	*r* = −0.891, *p* < 0.001^a^ ***p<0.003***	*r* = 0.899 *p* = 0.015 *N.S*.
Thalamus	*r* = −0.340, *p* = 0.215^a^ *N.S*.	*r* = −0.016, *p* = 0.98 *N.S*.
Midbrain	*r* = 0.014, *p* = 0.96^a^ *N.S*.	*r* = 0.360, *p* = 0.48 *N.S*.

Pearson partial correlations with age as a covariate; unadjusted significance level set at p < 0.05.

FDR Benjamini–Hochberg adjusted p-values presented in bold italics; significant at p < 0.05.

N.S., not significant after multiple comparisons.

a *Data for one participant is missing because of FreeSurfer preprocessing failure*.

### Relationship Between Left SLF and Language

**Figure 5:**
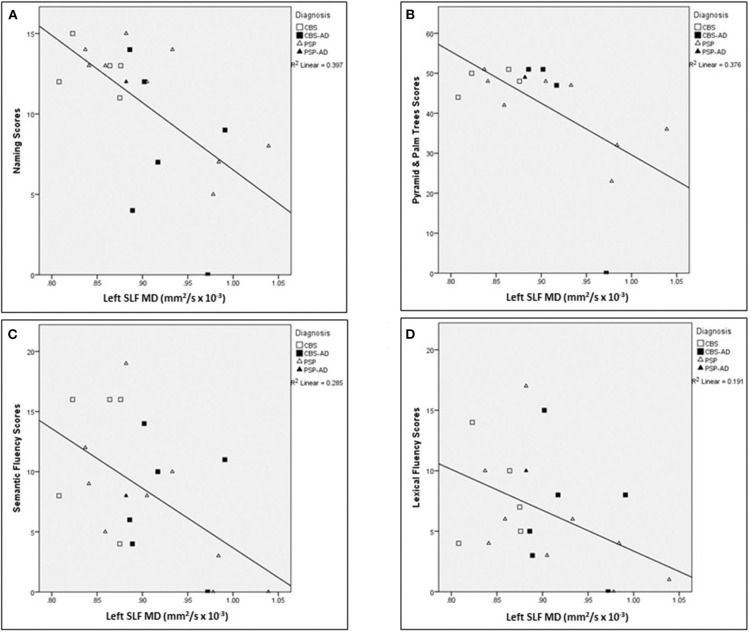
Associations between left SLF MD and language assessments across the whole cohort. Scatter plots presenting associations between left SLF MD (mm^2^/s × 10^−3^) and the following language assessments: **(A)** naming scores, **(B)** Pyramids and Palm Trees scores, **(C)** semantic fluency scores, and **(D)** lexical fluency scores. SLF, Superior longitudinal fasciculus; MD, Mean diffusivity.

For a summary of associations between left SLF white matter integrity measures in relation to language assessments across the whole cohort, controlled for age, see Table 6. The following associations between left SLF white matter integrity measures and language assessments were completed across the entire cohort. There was a significant negative correlation between naming score and the following left SLF white matter integrity measures: MD (*r* = −0.633, *p* = 0.008), AxD (*r* = −0.732, *p* < 0.007), and RD (*r* = −0.563, *p* = 0.022), controlled for age. There was a significant negative correlation between Pyramids and Palm Trees score and the following left SLF white matter integrity measures: MD (*r* = −0.792, *p* < 0.007), AxD (*r* = −0.910, *p* < 0.007), and RD (*r* = −0.707, *p* = 0.008), controlled for age. There was a significant negative correlation between semantic fluency score and the following left SLF white matter integrity measures: MD (*r* = −0.655, *p* = 0.008), AxD (*r* = −0.632, *p* = 0.008), and RD (*r* = −0.635, *p* = 0.008); controlled for age. There was a significant negative correlation between lexical fluency score and the following left SLF white matter integrity measures: MD (*r* = −0.507, *p* = 0.04) and RD (*r* = −0.512, *p* = 0.04), controlled for age. There was no significant correlation between sentence repetition scores and left SLF white matter integrity measures, controlled for age. There were no significant correlations between left SLF FA measures and any of the language assessments, controlled for age. Finally, there was no significant correlation between left SLF AxD values and lexical fluency scores, controlled for age. For visual representation of associations between left SLF MD and language assessments (see Figure 5).

**Table 6:** Associations between left SLF and language assessments across the whole cohort (mean ± standard deviation).

	**Left SLF measures (mm**^****2****^**/s); N** **=** **24**^****a****^			
	**FA**	**MD**	**AxD**	**RD**
**Language assessments**				
Naming	*r* = 0.285, *p* = 0.22 *N.S*.	*r* = −0.633, *p* = 0.003 ***p=0.008***	*r* = −0.732, *p* < 0.001 ***p<0.007***	*r* = −0.563, *p* = 0.010 ***p=0.022***
Pyramids and Palm Trees^b^	*r* = 0.409, *p* = 0.10 *N.S*.	*r* = −0.792, *p* < 0.001 ***p<0.007***	*r* = −0.910, *p* < 0.001 ***p<0.007***	*r* = −0.707, *p* = 0.002 ***p=0.008***
Sentence Repetition^c^	*r* = −0.003, *p* = 0.99 *N.S*.	*r* = −0.279, *p* = 0.26 *N.S*.	*r* = −0.363, *p* = 0.14 *N.S*.	*r* = −0.229, *p* = 0.36 *N.S*.
Semantic Fluency	*r* = 0.434, *p* = 0.056 *N.S*.	*r* = −0.655, *p* = 0.002 ***p=0.008***	*r* = −0.632, *p* = 0.003 ***p=0.008***	*r* = −0.635, *p* = 0.003 ***p=0.008***
Lexical Fluency	*r* = 0.390, *p* = 0.09 *N.S*.	*r* = −0.507, *p* = 0.022 ***p=0.04***	*r* = −0.442, *p* = 0.051 *N.S*.	*r* = −0.512, *p* = 0.021 ***p=0.040***

Pearson partial correlations with age as a covariate; unadjusted significance level set at p < 0.05.

FDR Benjamini–Hochberg adjusted p-values presented in bold italics; significant at p < 0.05.

N.S., not significant after multiple comparisons.

SLF, superior longitudinal fasciculus; FA, fractional anisotropy; MD, mean diffusivity; AxD, axial diffusivity; RD, radial diffusivity.

a Data for three participants are missing because of movement during DTI acquisition.

b Three participants refused the assessment.

c *Two participants refused the assessment*.

### Relationship Between Right SLF and Visuospatial Assessments

There were no significant correlations between any white matter integrity measures (FA, MD, AxD, and RD) of right SLF and VOSP position discrimination and number location assessments (all unadjusted *p* > 0.29) across the entire cohort, controlled for age.

### Relationship Between Fornix and Memory Assessments

There were no significant correlations between any white matter integrity measures (FA, MD, AxD, RD) of fornix and memory assessments (all unadjusted *p* > 0.34) across the entire cohort, controlled for age.

## Discussion

To our knowledge, this is the first study comparing parkinsonian syndromes (PSP and CBS) with and without AD biomarkers on measures of cognition, motor symptom burden, and neuroimaging findings of PET tau, white matter integrity, and volumes. Even though the FTLD-non-AD group was significantly older than the FTLD-AD group, there were no differences between groups on disease duration, severity of cognitive deficits as measured by CDR, and age of onset. The results about disease duration and age of onset might be inconclusive given high variability within each group and small sample size. Both groups had similar functioning on measures of language, executive function, memory, and visuospatial function. There were trends in FTLD-non-AD group for better functioning on the sentence repetition and digit forward assessments compared to the FTLD-AD group; however, these differences did not survive multiple comparisons. The results of this study showed significantly worse motor and parkinsonian symptom burden in the FTLD-non-AD group, compared to FTLD-AD. The higher motor disturbances and parkinsonian symptom burden were associated with smaller volumes in the basal ganglia and midbrain specifically across the FTLD-non-AD group; no such relationships were observed in the FTLD-AD group. The FTLD-AD group, in turn, had higher PET tau SUVR values on the ROI analysis across multiple frontal and temporal regions, and inferior parietal, compared to the FTLD-non-AD group. The FTLD-AD group had smaller volumes in AD-specific areas compared to the FTLD-non-AD group: inferior parietal, precuneus, and hippocampus. In turn, the FTLD-non-AD group had smaller midbrain volume compared to the FTLD-AD group. These results show that the presence of underlying AD pathology was associated with differences in phenotype of CBS and PSP syndromes with the AD biomarker positive group having less motor deficits and a trend that repetition is better in the non-AD group. Presumably, AD pathology as evidenced by AD biomarkers has a predilection for specific areas such as the inferior parietal, precuneus, and hippocampus while four repeat tau pathology has a predilection for the midbrain. Relative volumetric differences may be a potential biomarker to discriminate FTLD-non-AD from FTLD-AD. PET tau signal was increased globally across the brain regions in the FTLD-AD group, which was not specific to AD-related areas only.

The [18-F]AV1451 PET was significantly associated with measures of language, memory, and visuospatial function across the entire study cohort. The association between cognitive measures and [18-F]AV1451 PET tau is widely established in AD studies (43–45); however, the relationship between cognition and [18-F]AV1451 PET tau among the PSP and CBS cases is usually described as poor (45, 46). In this study, although the AD biomarker positive group was small, there was a significant relationship across the whole group between PET Tau SUVR in neuroanatomically relevant areas and cognitive function. This suggests that the ligand is detecting some abnormality even if it is not specific to the straight filaments seen in 4-repeat tau of PSP and CBD.

The voxel-based analysis of [18-F]AV1451 PET tau showed extensive areas of posterior and middle temporal signal increase in the FTLD-AD group, in comparison with FTLD-non-AD group and this pattern of uptake is consistent with previously published literature on distribution of [18-F]AV1451 ligand in AD (47). Aside from the cluster involving bilateral hippocampi, the rest of the clusters did not survive multiple comparisons. The reason could be that our study is underpowered due to the small sample size. Because of the small sample size, the comparisons between [18-F]AV1451 PET tau and CSF measures were completed across the entire cohort. Cortical gray matter was chosen as a global measure of PET tau signal, and it was significantly associated with CSF measures of t-tau, Aβ42, and ATI; however, there was no significant relationship with p-tau. Our results confirm previous reports stating a good correlation between CSF t-tau and [18-F]AV1451 PET (48, 49), but we found less robust relationship between p-tau and [18-F]AV1451 compared to others. Even though there is high concordance between CSF and [18-F]AV1451 measures, CSF p-tau abnormality seems to precede [18-F]AV1451 PET tau positivity and marks the early stages of underlying AD pathology (49). However, at the later stages of disease where cognitive decline has become apparent, [18-F]AV1451 PET tau has proven to be a significantly better diagnostic tool of AD than CSF measures, hippocampal atrophy, or temporal cortices thickness (50).

The literature on biomarkers to detect the differences between FTLD-non-AD and FTLD-AD is quite limited and mostly comes from pure cohorts of either CBS or PSP cases (not heterogeneous cohorts as implemented in this study) or from single case studies. PET with [18-F]AV1451 tracer has been established as a biomarker of tau accumulation in the form of AD's paired helical filament and showed good correlation with Braak stages of AD and post-mortem assessments (51). The PET [18-F]AV1451 tracer has not been reported suitable to detect the straight filament tau of CBD and PSP, and showed poor correlation to post-mortem studies in these cohorts (52–54). From previous reports, increased PET tau tracer retention in CBS cases without underlying AD pathology was noted in the motor cortex, corticospinal tract, and basal ganglia contralateral to the affected body side, while the tracer retention pattern in AD is concentrated mostly in temporal and parietal cortices (55). Studies using the same PET tau tracer in PSP cases reported increased retention in basal ganglia, but not cortical regions (52). Our study shows similar findings of increased tracer uptake in dorsal caudate, thalamus, and brainstem (the regions affected by pathology in parkinsonian syndromes), and these findings were similar in both FTLD-non-AD and FTLD-AD groups and did not differentiate one group from another. The increased PET tau SUVR values in the FTLD-AD group were seen across multiple frontal and temporal regions, as well as inferior parietal, and the signal was not restricted to AD-specific regions. This goes in hand with previous literature stating that PET [18-F]AV1451 tracer is better suited for detecting tau in AD. The [18-F]AV1451 PET tau tracer is a biomarker that can discriminate parkinsonian syndromes with and without underlying AD pathology, but does not tell us about the extent of AD pathology in the FTLD-AD group.

The decreased focal volumes in the FTLD-AD group of our study support previous findings in cohorts with clinical presentation of CBS with evidence of AD pathology having a higher degree of volume loss in the temporoparietal regions, compared to CBS due to non-AD pathologies (5). Even though our FTLD-non-AD cohort has PSP cases in addition to CBS, cortical and sub-cortical volume loss was not reported to be different across parkinsonian syndromes (56), and therefore it is unlikely that our results were affected by the heterogeneity of our cohort. Midbrain atrophy was previously reported to be a hallmark of clinical PSP, but not the pathological diagnosis of PSP without the clinical PSP presentation (57). Also, clinically diagnosed CBS and PSP cohorts had similar levels of midbrain atrophy upon comparison (58). Our study also included clinically diagnosed CBS and PSP cases, and the FTLD-non-AD group had a higher degree of midbrain atrophy compared to the FTLD-AD group. These results imply that AD and 4-repeat pathology have a predilection for different areas.

One study comparing CBS-non-AD and CBS-AD across neuropsychological tests concluded similar levels of memory impairment and attention deficits in both groups (59). When comparing CBS-AD cases to AD patients with typical amnestic syndrome (AD-AS), it was reported that language problems were more frequent in the CBS-AD group, while memory impairment was the hallmark of the AD-AS group. As expected and in contrast to AD-AS, CBS-AD had prominent motor deficits (60). Since language impairments are an early and persistent problem in CBS and PSP (61), our results support previous literature as in our study too, the FTLD-non-AD and the FTLD-AD groups had similar language and cognitive functioning; however, motor impairments were worse in the FTLD-non-AD group. Due to similar levels of language impairment across FTLD-non-AD and FTLD-AD groups, the two groups were combined, and as expected, decreased white matter integrity in the left SLF tract (implicated in language) corresponded to a higher degree of impairment on language assessments (Figure 2). This suggests similar degrees of underlying pathological changes in white matter integrity across both groups, and these white matter changes are at least partially contributing to the widespread language impairments in CBS and PSP.

There are a number of limitations in the current study, including the small cohort size, which decreases the power of our analyses. The participants' head movement can always introduce artifacts into the PET data affecting the results, despite completing the PET frame realignment in order to correct for motion. Participants with CBS and PSP diagnoses are not equally represented in both groups, which could have affected the results. Due to the absence of post-mortem assessment results, there is no way of knowing the dominant or exclusive underlying pathological diagnoses, and the diagnoses were made based on the clinical presentation and progression of the symptoms. Patients lacking markers for AD probably had predominant 4R tau pathology, although CBS patients could have had other non-tau non-AD pathology. Patients with AD markers may have had only AD pathology or a combination of AD plus other pathologies, especially 4R tauopathy. In addition, the absence of a reliable control group did not allow us to draw any conclusions about the degree of pathological changes that either one of our study groups had in comparison to healthy aging. Finally, we could not assess the relationship between the neuropsychological measures, CSF markers, and [18-F]AV1451 PET tau in FTLD-non-AD and FTLD-AD groups separately due to small sample size.

Overall, our results showed that PET with [18-F]AV1451 tau-specific tracer can discriminate FTLD-non-AD and FTLD-AD cohort by its high specificity to paired-helical filaments seen in AD, and is associated with a higher global PET signal in the FTLD-AD group. Volumetric analysis seems to provide evidence of pathological vulnerability. There is decreased volume in areas implicated in classic AD in the CBS-AD and PSP-AD groups (hippocampus and precuneus), which implies that there are AD pathology vulnerable areas, as this group was younger than the non-AD group so less atrophy might have been expected. The presence of underlying AD pathology in parkinsonian syndromes is associated with a different phenotype of the presenting illness, with increased motor disturbances in the FTLD-non-AD group, while the level of cognitive functioning is the same irrespective of the presence of AD pathology.

## Data Availability Statement

The datasets generated for this study are available on request to the corresponding author.

## Ethics Statement

The studies involving human participants were reviewed and approved by University Health Network and Center for Addiction and Mental Health. The patients/participants provided their written informed consent to participate in this study.

## Author Contributions

AV acquired the data, analyzed the data, interpreted the data, and drafted the manuscript for intellectual content. FT analyzed and interpreted the data. NM, CA, and KM had major roles in data acquisition. SH interpreted the data and revised the manuscript for intellectual content. CB analyzed and interpreted the data. DT-W, AL, SF, and ES had major roles in data acquisition. PR interpreted the data and revised the manuscript for intellectual content. MT had a major role in acquisition of data, interpreted the data, and drafted and revised the manuscript for intellectual content. All authors contributed to the article and approved the submitted version.

## Conflict of Interest

The authors declare that the research was conducted in the absence of any commercial or financial relationships that could be construed as a potential conflict of interest.
